
JOURNAL INFORMATION
==============================
NLM Title Abbreviation: Behav Neurol
Journal ID: Behav Neurol
NLM Journal ID: 8914585
Journal ID: BN

Behavioural Neurology

ISSN: 0953-4180
EISSN: 1875-8584
Publisher: Wiley

ARTICLE INFORMATION
==============================
PMCID: PMC5434447
PMID: 21422569
DOI: 10.3233/BEN-2010-0309
Article ID: 741257
Article version: 1
Subjects: Research Article

Feature-Based Processing of Personally Familiar Faces in Prosopagnosia: Evidence from Eye-Gaze Contingency

Van Belle Goedele 1 *
Lefèvre Philippe 1
Laguesse Renaud 1
Busigny Thomas 1
de Graef Peter 2
Verfaillie Karl 2
Rossion Bruno 1
1 Université Catholique de Louvain Louvain-la-Neuve Belgium
2 Katholieke Universiteit Leuven Leuven Belgium
*Goedele Van Belle: goedele.vanbelle@uclouvain.be
Print publication date: 2010
Electronic publication date: 2011 Mar 7
Volume: 23
Issue: 4
First page: 255
Last page: 257
Received 2011 Jan 31; Accepted 2011 Jan 31
Copyright: Copyright © 2010 Hindawi Publishing Corporation and the authors.
Copyright year: 2010
License: This is an open access article distributed under the Creative Commons Attribution License, which permits unrestricted use, distribution, and reproduction in any medium, provided the original work is properly cited.
License URL: https://creativecommons.org/licenses/by/3.0/

==============================
Behavioural Neurology 23 (2010) 255–257
DOI 10.3233/BEN-2010-0309
IOS Press

### Feature-based processing of personally familiar faces in prosopagnosia: Evidence from eye-gaze contingency

Goedele Van Belle[a,*], Philippe Lefèvre[a], Renaud Laguesse[a], Thomas Busigny[a], Peter de Graef[b], Karl Verfaillie[b] and Bruno Rossion[a]
[a] *Université Catholique de Louvain, Louvain-la-Neuve, Belgium*
[b] *Katholieke Universiteit Leuven, Leuven, Belgium*

### 1. Introduction

How familiar and unfamiliar faces are perceived remains largely unknown. Two views have dominated this field of research. On the one hand, recordings of eye fixations on faces [10] and response classification experiments [2] suggest that a face is processed in terms of its individual components, or facial features (mouth, eyes, nose, ...), a strategy called *analytical processing*. On the other hand, there is strong behavioral evidence for interdependence in the processing of different features of a face [6,7], rather supporting *holistic processing* of the face [7]. According to the latter holistic view, facial features are simultaneously perceived and integrated into a single representation, so that the perceptual field is that of the whole face.

To shed light on this issue, in two recent studies, we recorded eye movements in a neurological patient [5] suffering from a selective impairment in face recognition (acquired prosopagnosia). Previously, we showed that (1) PS fixates exactly on each of the main features of the face (mouth, left eye, right eye), contrary to normal observers who fixate mainly centrally on the top of the nose, around the geometric centre of the face [3] (Fig. 1a). Moreover (2), an original gaze-contingent stimulus presentation method [9] applied to an unfamiliar face discrimination task led us to demonstrate that, contrary to normal observers, PS' perceptual field appears to be limited to one central feature fixated at a time [8] (Fig. 1b). These observations indicate that prosopagnosia prevents processing the multiple elements of a whole face simultaneously, and thus that this ability is a key aspect in human face recognition expertise. Here, we extend these observations by testing the same patient with eye gaze contingency while she attempts to identify a large set of personally familiar individuals from their face.

*Corresponding author: Goedele van Belle, Université Catholique de Louvain (UCL), Faculté de Psychologie et des Sciences de l'Education (PSP), Unité de Cognition et Développement (CODE), Place du Cardinal Mercier, 10, B-1348 Louvain-la-Neuve, Belgium, Tel.: +32 10 47 92 60; Fax: +32 10 47 37 74; E-mail: goedele.vanbelle@uclouvain.be.

### 2. Methods

PS (born 1950) had brain damage in 1992, with a main lesion in the right inferior occipital cortex. Difficulty at identifying faces is her only complaint, and object recognition is preserved. She was tested in numerous behavioural and neuroimaging studies over the past 10 years (see e.g. [1,5]). Despite her prosopagnosia, she still works as a kindergarten teacher, so that she is familiar with a large set of young children and uses many cues to recognize them in everyday life (3–4 years old; see [3] for details).

Here we took high quality photographs of 29 of these children's faces (2009). Extrafacial features were cropped. Stimuli were displayed using Presentation, on a 22" Sony Trinitron monitor at a viewing distance

ISSN 0953-4180/10/$27.50 © 2010 – IOS Press and the authors. All rights reserved

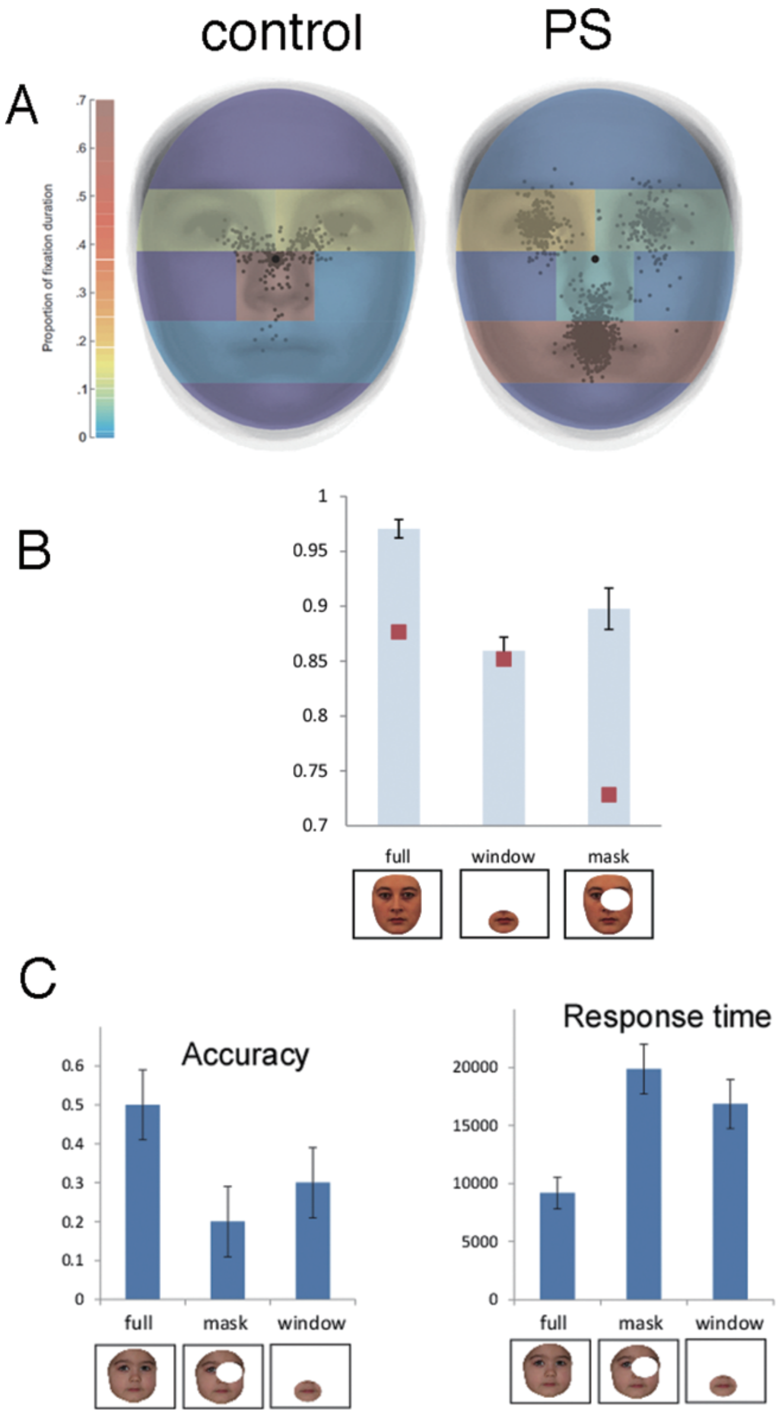

Fig. 1. **A**. Adapted from Orban de Xivry et al. [3]: fixation location of a normal observer and PS when identifying personally familiar faces. Note that PS fixates exactly on each diagnostic feature, with a very large proportion of fixations on the mouth (about 60%). **B**. Accuracy results of the eye-gaze contingency study of Van Belle et al. [8], showing that the prosopagnosic patient PS' performance (the square dots) is much more affected in the mask than in the window condition, contrary to normal observers (the bars). **C**. In the present short study, gaze contingency was used with pictures of personally familiar children for the patient PS, who performed again lower with a window than a mask (increase of errors ad RTs; accuracy level is based on an identification task with a chance level of 1/29).

of 58 cm with a spatial resolution of 1280 by 1024 pixels and a refresh rate of 80 Hz. Height of the faces was 12 degrees of visual angle. In addition to the full view condition, faces could be presented with either a gaze-contingent *window* or a gaze contingent *mask* (Fig. 1B and 1C). A window revealed only the momentarily fixated feature of the stimulus, forcing PS to use an analytical processing strategy (one feature at a time). A gaze-contingent mask, on the contrary, constantly covered the centre of gaze, containing the most detailed, high-resolution information and therefore interfered with an analytical processing strategy, forcing PS to try to rely on holistic processing. The size of the mask and window was 8.5 by 6.5 degrees of vision.

Both stimulus display and response registration were handled by an Intel Centrino vPro. Eye movements were registered with an SR Research Eyelink 1000 remote eye tracker at a sampling rate of 250 Hz and with gaze position error smaller than $0.5^{\circ}$. Head movement was restricted by a chin and head rest.

A drift correction was followed by a fixation cross to the left of the face area. Upon fixation of the cross, the face of one of the children appeared, visible through one of the three viewing conditions. PS could freely explore the face and hit the space bar as soon as she thought she could identify the child. Then a list of all names appeared and PS responded by simultaneously fixating the recognized expression and hitting the space bar a second time. Accuracy rates, exploration duration (ms), number of saccades and fixations were measured.

PS completed 2 blocks of 45 trials each. Viewing conditions were randomized within the blocks.

### 3. Results and discussion

PS' level of performance with faces presented in full view was at 50%, so she recognized half of the children from their (cropped) faces (chance level is 1/29). This performance dropped in the two gaze-contingent conditions, to 30% in the window condition (statistically lower than full view condition: $X^2$ test of two proportions, $p = 0.06$) and 20% in the mask condition ($p = 0.009$). The mask and window condition did not differ significantly from each other ($p = 0.18$). PS was slower, made more fixations and saccades in the two gaze-contingent conditions than when the faces were presented at full view (Fig. 1C; full vs. window or full vs. mask, all ps $< 0.001$). However, in line with accuracy scores, she was also marginally slower in the mask than in the window condition ($p = 0.15$) and made more and longer fixations ($p = 0.07$ for both measures). We were not able to test normal controls in this task, given that PS was the only person who had been exposed to this large set of children faces and was available for the experiment. Nevertheless, these results are in line with observations made on the same patient when matching unfamiliar faces with gaze-contingency [8] (Fig. 1B). In that study, normal observers presented an opposite response profile than the patient, performing better in the mask than in the window condition. Moreover, a recent study with normal observers and familiar faces also show that identifying faces is generally faster in the mask than in the window condition (Ramon et al., submitted), unlike what is found for PS here.

Altogether, these observations support the view that the prosopagnosic patient PS has difficulties with processing the whole of an individual face, and that she rather relies on individual facial features in order to identify faces. Hence, being able to process an individual face holistically appears to be a key element in human face perception expertise.

### References

[1] T. Busigny and B. Rossion, Acquired prosopagnosia abolishes the face inversion effect, *Cortex* **46** (2010), 965–981.

[2] N.D. Haig, How faces differ – a new comparative technique, *Perception* **14** (1985), 601–615.

[3] J.-J. Orban de Xivry, M. Ramon, P. Lefèvre and B. Rossion, Reduced fixation on the upper area of personally familiar faces following acquired prosopagnosia, *Journal of Neuropsychology* **2** (2008), 245–268.

[4] M. Ramon, G. Van Belle, P. Lefèvre and B. Rosion, (submitted). Holistic perception of fa, miliar and unfamiliar afces: an eye-gaze contingency study.

[5] B. Rossion, R. Caldara, M. Seghier, A.-M. Schuller F. Lazeyras and E. Mayer, A network of occipito-temporal face-sensitive areas besides the right middle fusiform gyrus is necessary for normal face processing, *Brain* **126** (2003), 2381–2395.

[6] J. Sergent, An investigation into component and configural processes underlying facial perception, *British Journal of Psychology* **75** (1984), 221–242.

[7] J.W. Tanaka and M.J. Farah, Parts and wholes in face recognition. The Quarterly Journal of Experimental Psychology. A, *Human Experimental Psychology* **46** (1993), 225–245.

[8] G. Van Belle, P. de Graef, K. Verfaillie, T. Busigny and B. Rossion, Whole not hole: expert face recognition requires holistic perception, *Neuropsychologia* **48** (2010), 2609–2620.

[9] P.M.J. van Diepen, P. De Graef and J. Van Rensbergen, On-line control of moving masks and windows on a complex background using the ATVista videographics adapter, *Behavior Research Methods, Instruments, and Computers* **26** (1994), 454–460.

[10] A.L. Yarbus, Eye movements during perception of complex objects, in *Eye Movements and Vision*, L.A. Riggs, ed., Plenum Press, New York, 1967.
